# Limited overall impacts of ectomycorrhizal inoculation on recruitment of boreal trees into Arctic tundra following wildfire belie species-specific responses

**DOI:** 10.1371/journal.pone.0235932

**Published:** 2020-07-09

**Authors:** Rebecca E. Hewitt, F. Stuart Chapin, Teresa N. Hollingsworth, Michelle C. Mack, Adrian V. Rocha, D. Lee Taylor

**Affiliations:** 1 Institute of Arctic Biology, University of Alaska Fairbanks, Fairbanks, Alaska, United States of America; 2 Center for Ecosystem Science and Society, Northern Arizona University, Flagstaff, Arizona, United States of America; 3 US Forest Service, Pacific Northwest Research Station, Boreal Ecology Cooperative Research Unit, Fairbanks, Alaska, United States of America; 4 Department of Biological Sciences and the Environmental Change Initiative, University of Notre Dame, Notre Dame, Indiana, United States of America; 5 Department of Biology, University of New Mexico, Albuquerque, New Mexico, United States of America; University of California Berkeley, UNITED STATES

## Abstract

We tested whether post-fire seedling establishment of common boreal tree and expanding shrub species at treeline and in Arctic tundra is facilitated by co-migration of boreal forest mycorrhizal fungi. Wildfires are anticipated to facilitate biome shifts at the forest-tundra ecotone by improving seedbed conditions for recruiting boreal species; at the same time fire alters the composition and availability of mycorrhizal fungi critical to seedling performance. To determine the role of root-associated fungi (RAF) in post-fire seedling recruitment and future biome shifts, we outplanted four dominant boreal tree and shrub species inoculated with one of three treatments at treeline and in tundra: burned boreal forest, unburned boreal forest, or a control treatment of sterilized inoculum. We compared survivorship, growth, and physiological performance of the seedlings in relation to mycorrhizal inoculum treatment and among host species, characterized the RAF communities based on ITS-rDNA sequencing of individual root tips sampled from surviving seedlings, and tested for correlations between RAF composition and the inoculation treatments, host species, and duration of the experiment. We explored correlations between RAF composition and seedling metrics. Both live and sterile autoclaved inoculation treatments had similar effects on seedling survivorship and growth for all species. RAF composition did not vary by treatment, suggesting that most colonization was due to local fungi. However, seedling traits and growth were correlated with RAF species composition, colonization, and the relative abundance of specific RAF taxa. *Picea* sp. performance in particular showed strong co-variation with RAF metrics. Our results suggest that mycorrhizal co-migration is not a primary limiting factor to boreal seedling recruitment because the experimental provision of inoculum did not affect seedling recruitment; yet, RAF did influence seedling performance, particularly resident RAF at treeline and in tundra, suggesting that mycorrhizal fungi are important to vegetation processes at the treeline-tundra ecotone.

## Introduction

Establishment of boreal tree species and shrubs beyond their current range in Arctic tundra may impact climate through increases in carbon storage capacity and reductions in albedo [1, 2]. However, the major controls on colonization of the tundra remain poorly understood. Although research indicates that warming alone correlates strongly with tundra greening [3–5], warming experiments do not yield clear positive effects of warmer air temperature on germination, growth, and survival of tree species in tundra. Contrary to expectations, experimental warming has even proved detrimental for some species [6, 7]. Consequently, some have suggested that plant access to belowground resources [6], potentially via root-associated fungi (RAF), may be an important driver of vegetation transitions as the climate warms [8].

Limited tree-compatible RAF beyond current treeline could inhibit tree and shrub seedling establishment. Outside of the Arctic, research shows that low availability of RAF can limit seedling establishment [9, 10]. Boreal trees and shrubs are obligately ectomycorrhizal, relying on ectomycorrhizal fungi (EMF) for nutrient uptake [11], and the Arctic treeline ecotone represents a transition from EMF to ericoid mycorrhizal (ERM) dominance [12, 13]. Therefore, treeline and tundra habitats may represent conditions where seedling-compatible EMF are limited beyond the rhizosphere of low-density EMF hosts [14, 15]. Conversely, the development of novel vegetation communities can be facilitated by the co-migration of mycorrhizal symbionts [16, 17]. Taken together this suggest a potentially important role of plant-fungal interactions for Arctic vegetation change.

Wildfire is expected to facilitate shrub expansion in tundra [18] and biome shifts at the forest-tundra ecotone [19] by producing favorable recruitment conditions and thus shaping successional trajectories. In the boreal forest after low and moderate severity fires, historically, spruce recruits, and a self-replacement trajectory ensues. After high severity fires, deciduous species preferentially recruit shifting the successional trajectory to deciduous dominance [20]. In tundra increased growth and reproduction of deciduous tall shrubs, like *Alnus viridis* (Chaix) DC, in burned sites [21] indicates that fire may support the expansion of shrubs. At treeline and in tundra low density EMF hosts, such as *Betula nana* L. and *Salix* sp., support generalist fungi [15, 22, 23], and thus the fungi present in these habitats should be compatible with recruiting shrubs and deciduous and coniferous tree seedlings. As such, fire effects on fungal communities at treeline and in tundra may have important impacts on successional trajectories [15, 24, 25], particularly if recruiting seedlings vary in their sensitivity to variation in the post-fire fungal community.

Wildfire incurs a strong filter on the symbiotic fungal community. Fire-severity affects the abundance of surviving host plants, the degree of soil heating and combustion, and thus the legacy of pre-fire, often ‘late-stage’ RAF [26]. Shifts in fungal community structure and a reduction in mycorrhizal colonization levels after fire [27–29] can influence seedling performance [30] and persist for the duration of the recruitment phase [29, 31]. Several studies have shown strong differences in inoculum potential and composition between burned and unburned forests [27, 32]. Because of the taxon-specific costs and benefits of RAF [33–36], variation in RAF inoculum potential and composition after fire could have strong effects on seedling performance. Considering the potential for treeline and tundra to have limited inoculum potential due to low EMF host plant abundance, increased wildfire activity at treeline and in tundra may represent conditions where RAF are extremely limited and plant-fungal associations have a strong effect on recruitment and succession.

Our study aimed to address whether post-fire seedling establishment of common boreal tree species and expanding shrubs is limited by mycorrhizal associations. We tested whether seedling survival, growth, and performance were impacted by inoculation with RAF communities sourced from burned or unburned boreal forest. Our research sought to address the following questions:

1. Does the provision of boreal forest mycorrhizal inoculum facilitate tree and shrub seedling establishment at and beyond treeline?
2. Are host species equally responsive to RAF inoculum availability, colonization, and composition?
3. Does the species composition and degree of colonization by RAF inoculum affect seedling performance?

## Materials and methods

### Site description

We planted RAF-inoculated boreal tree and shrub seedlings at two northern Alaskan sites representing current and future treeline conditions (Table 1). At latitudinal treeline we planted seedlings at Finger Mountain (66° 20’ 28.41” N, 150° 27’ 14.32” W) five years after it burned. Unburned forest cover at Finger Mountain was dominated by acidic black spruce (*Picea mariana* (Mill.) Britton, Sterns & Poggenb) communities [37] with patches of deciduous broadleaf trees, mainly trembling aspen (*Populus tremuloides* Michx.) and Alaskan paper birch (*Betula neo-alaskana* Sarg.). Treeline was a mix of white spruce (*Picea glauca* Moench) and black spruce. Tundra is dominated by ERM-hosting ericaceous shrubs, EMF-hosting *B*. *nana* and *Salix* sp., graminoids, and feather mosses on rocky substrates. The Finger Mountain fire created a moderate-severity burn scar; however, due to the rocky soils with thin pre-fire soil organic layers, the post-fire soil environment at our site was similar to a high-severity burn. Inside the burn scar the mean seedling density was 0.13 ± 0.03 S.E. m^2^, the majority of which were *P*. *tremuloides* (0.10 ± 0.03 S.E. m^2^) not *Picea* sp. (0.03 ± 0.02 S.E. m^2^). Close to Finger Mountain, mean annual temperature is -2.1°C and mean annual cumulative precipitation is 477 mm (NRCS SNOTEL Gobblers Knob 2007–2018). Approximately 200 km north of current Arctic treeline we planted seedlings in the burn scar of the Anaktuvuk River Fire (ARF, 68° 59' 37.83'' N, 150° 18' 19.31'' W) two years after the fire. The ARF burned a region characterized by rolling uplands of shrubby tussock tundra underlain with continuous permafrost. The outplant site in the ARF was severely burned and there were no naturally occurring tree or shrub seedlings within the outplanting site. Close to the ARF, mean annual temperature is -5.2°C and mean annual cumulative precipitation is 331 mm (NRCS SNOTEL Imnaviat Creek site 2007–2018). Site access was permitted by Bureau of Land Management through the Bonanza Creek Long Term Ecological Research (LTER) program for Finger Mountain and the Arctic LTER for the Anaktuvuk River Fire.

**Table 1:** Site characteristics of outplanting locations at Arctic treeline and tundra. EMF = Ectomycorrhizal fungi.

Location	Site	Latitude, longitude	Natural seedling establishment m^-2^ ± S.E.	Year of burn	EMF host plants
Arctic treeline	Finger Mountain	66° 20’ 28.41” N, 150° 27’ 14.32” W	0.13 ± 0.03	2004	*Picea mariana*, *Picea glauca*, *Populus tremuloides*, *Betula neo-alaskana*, *Alnus viridis*, *Betula nana*, *Salix sp*.
Arctic tundra	Anaktuvuk River	68° 59' 37.83'' N, 150° 18' 19.31'' W	--	2007	*Betula nana*, *Salix* sp.

### Seedling preparation

We germinated seeds of *P*. *mariana*, *P*. *glauca*, *Alnus viridis*, and *B*. *neo-alaskana* collected in interior Alaska at Washington Creek (65° 6' 47.58" N, 148° 17' 12.53" W) and Galena (64° 43' 60.00" N, 156° 55' 39.00" W). Seed was frozen at -20°C until germination. To remove any contaminating microorganisms before inoculation we surface-sterilized seeds in a five percent bleach and liquinox solution. Seeds were placed in sterile petri dishes on autoclaved filter paper and kept moist with RO water. Seedlings were planted 3–5 weeks after germination into conetainers filled with an autoclaved 3:1:1 mix of peat:vermiculite:perlite.

We inoculated seedlings with one of three inoculum treatments: burned forest, unburned forest or sterile autoclaved inoculum. Inoculum consisted of mixed root tips harvested from focal seedling species (*P*. *mariana*, *P*. *glauca*, *A*. *viridis*, and *B*. *neo-alaskana* and also *P*. *tremuloides*) growing in burned and unburned boreal forest sites at Caribou Poker Creek Watershed (CPCRW, 65° 8' 24.17" N, 147° 27' 14.90" W) outside Fairbanks, Alaska. The wildfire that burned forest sites in CPCRW occurred five years before inoculum collection. We washed and excised fine root segments with healthy ectomycorrhizal root tips from seedlings. Sampled roots were chopped into 0.5 cm sections and pooled by species and treatment (burned, unburned, or sterile control). The sterile autoclaved inoculum consisted of equal parts of root tips from each host species from both burned and unburned forest that were pooled and autoclaved. Subsamples of burned and unburned inoculum from each host plant were frozen at -80°C until further molecular characterization of the inoculum community.

To inoculate each seedling we exposed the lateral roots by clearing away some of the potting mix and placing several root tips (~ 0.1 ml) from each tree species adjacent to living roots of the seedling. Sterile autoclaved inoculum was applied to seedlings identically to the burned and unburned live inoculum. Before planting the seedlings, we measured their height and hardened them off by moving them outside the greenhouse to adjust to the outside light and temperature regime for two weeks. We also collected foliar tissue for an initial time point isotopic analysis from a set of seedlings that were not outplanted. One month after inoculation seedlings were transported to the Arctic treeline (n = 192) or tundra (n = 192) and outplanted along 16 m transects at 1 m intervals at both sites.

### Field methods

In the two years after inoculation, we visited both outplant sites in July during peak biomass. Each visit we assessed seedling survival and growth, and then harvested half of the seedlings to assess mycorrhizal composition. As such, half of the seedlings were harvested one year after inoculation and outplanting and half of the seedlings were harvested two full years after inoculation and outplanting. Each year we also measured instantaneous maximum photosynthesis and respiration on surviving seedings and calculated carbon use efficiency (CUE). Due to the small size of some of the seedlings we were not able to measure gas exchange for all surviving seedlings. We measured maximum photosynthesis with five readings at 1500 μmols of light and respiration with five readings in the dark at a temperature of 20°C using a LI-COR 6400 (LI-COR, Inc., Lincoln, NE, USA). Measurements were then averaged and CUE was calculated as [1- (respiration/photosynthesis)]. The gas exchange calculations were adjusted for differences in leaf area by scanning leaves [600 dpi on an HP ScanJet 4570c scanner with HP PrecisionScan Pro software] and measuring leaf area of the scanned images with ImageJ [38].

### Laboratory methods

After harvesting seedlings we separated roots from shoots. Roots were stored at 4°C and processed within five days. Each root system was gently rinsed with RO water to remove soil, and clean roots were placed in RNAlater (Ambion, Inc., Austin, Texas, USA) until further molecular analyses (described below). For seedlings harvested two years after planting, we separated leaves from stems, dried tissues at 60°C for 48 hours, ground foliar samples, and ran them for Elemental Analysis- Isotope Ratio Mass Spectrometer (IRMS, Delta Advantage, Thermo Fisher Scientific, Waltham, MA, USA) coupled to an Elemental Combustion Analyzer (Costech ECS4010, Valencia, CA, USA). We followed the same steps for processing foliar samples collected from the subset of seedlings that were hardened off but not outplanted and served as baseline isotope values.

### Molecular methods

#### Cloning and sequencing of inoculum

To determine the fungal composition of the burned and unburned forest inoculum we ground lyophilized inoculum in lysis buffer with a motorized sterile pestle (Kontes, Rockwood, Tennessee, USA). DNA was extracted from two replicates from each sample (host plant x treatment) using the DNEasy Plant Mini Kit (*QIAGEN* Inc., Valencia, California, USA) according to the manufacturer’s instructions. Genomic DNA was amplified using 50 μM of the forward primer ITS1F (CTTGGTCATTTAGAGGAAGTAA; [39] and reverse primer ITS4 (TCCTCCGCTTATTGATATGC; Amersham PCR beads (GE Healthcare Bio-Sciences, Marlborough, MA, USA) and 5 μl of genomic DNA diluted 100x. We mixed three replicate PCR reactions for each host plant and treatment combination. Reaction mixes were prepared in 0.2 ml tubes and thermocycled in an MJ Research PTC-225 thermal cycler as follows: 96°C for 2 min, 26 cycles of 94°C for 30s, 55°C for 40s, then 72°C for 1 min, followed by 72°C for 10 min. When we gel checked the PCR products we found that each PCR product produced bands with comparable fluorescence and we therefore pooled 10 ul of each sample into a pooled burned and unburned forest inoculum sample. This was shipped on wet ice overnight to Functional Biosciences Inc., Madison, WI for cloning and Sanger sequencing. Samples were cloned using the TOPO TA Cloning^®^ Kit for Sequencing (Invitrogen, Carlsbad, CA, USA). Using a blue-white screen, colonies that had an insert present were picked and grown in one ml of TB in a 2.2 ml block. Plasmids were extracted and purified using the Promega SV9600 Plasmid DNA purification system (Promega Corporation, Madison, WI, USA). Samples were amplified using the primers M13F (TGT AAA ACG ACG GCC AGT) and M13R (CAG GAA ACA GCT ATG ACC) and sequenced using Big Dye Terminator v3.1 using standard cycling conditions and run on an ABI 3730xl DNA analyzer (Applied Biosystems, Carlsbad, CA, USA).

#### Mycorrhizal colonization and community structure on seedling root tips

Each seedling root system was cut into four cm root segments and floated in nanopure water. For seedlings harvested two years after inoculation, we assessed the proportion of root length colonized by RAF using the point intercept method [40]. To characterize the RAF community, we randomly selected ten root tips from each seedling. All root tips were separately lyophilized and ground in lysis buffer with a motorized sterile pestle (Kontes, Rockwood, Tennessee, USA). DNA was extracted from each root tip using the DNEasy Plant Mini 96-Plate Kit (*QIAGEN* Inc., Valencia, California, USA) according to the manufacturer’s instructions, except for one modification, the addition of a Proteinase K incubation to denature RNAlater-protein-DNA complexes as suggested in Bent and Taylor [41]. Genomic DNA from all samples was amplified using ITS1F and ITS4, as above, except that 35 rather than 26 cycles were carried out. PCR products were gel checked and the individual root tips with bright bands were shipped on wet ice overnight to Functional Biosciences Inc., Madison, WI for Sanger sequencing.

### Bioinformatics

Sanger sequences were trimmed, quality filtered, and grouped into operational taxonomic units (OTUs), synonymous with RAF taxa, as follows. Initial end-trimming (error rate below 0.1 in a 25 base window for both ends) and assembly of paired end reads (where available) were carried out in Codoncode Aligner 4.03. Sequences with fewer than 200 high quality base calls (phred >20) were deleted and all remaining sequences were exported in fastq format. Next, a more stringent end-trimming was carried out using FASTQ Quality Trim program in Galaxy [42], with window sizes of 5, 20 and 40 and a phred threshold of 20. Sequences were converted to fasta format in Galaxy then aligned using Muscle [43] in AliView [44] to identify and manually remove primer sequences. We then used QIIME 1.9 [45] to cluster sequences into OTUs via open-reference with the USEARCH method [46], a 95% sequence identity threshold, and the UNITE sh_refs_qiime_ver7_97_s_20.11.2016 database [47]. Singletons were kept at this step (min_otu_size 1). Representative sequences for each OTU were submitted to the RDP Naïve Bayesian classifier using the Warcup 2 ITS training set to estimate OTU taxonomies [48]. We also searched GenBank using discontiguous mega-BLAST, with uncultured sequences excluded, to confirm, and in some cases, improve identifications produced by RDP. OTU trophic guilds were assigned based on FunGUILD assignments and investigator knowledge. All sequences are deposited in GenBank under accession numbers MT527965—MT528153. For analyses testing the relationship between fungal composition and plant performance (gas exchange, foliar traits, biomass, growth) we reduced the species matrix to include only putatively RAF taxa, excluding known pathogens and saprotrophs from the dataset.

### Statistics

#### Inoculation experiment

All analyses were conducted in R version 3.6.1 [49]. We tested whether mycorrhizal colonization varied with host species or inoculation treatment using a linear model. We evaluated specific contrasts of the response of each host species to the mycorrhizal inoculation treatment and across species receiving the same inoculation treatment with the emmeans package [50] using a post hoc Tukey p-value adjustment.

To test whether survivorship at Arctic treeline and tundra varied by host plant species, inoculation treatment, and duration of outplanting we used logistic regression. We built separate models for each outplanting location and year with species and inoculation treatment as predictors and survivorship as the response. In each case, the interaction between species and inoculation treatment was tested, found to lack explanatory power, and the additive model was reported. We evaluated contrasts of factors and accounted for multiple comparisons with the Tukey p-value adjustment.

To test whether growth among species varied by inoculation treatment at Arctic treeline and tundra we used a linear model. We calculated growth by scaling seedling height at the time of harvest (year one or two) minus the initial height before outplanting. Many seedlings shrank between measurements due to apical damage and resprouting and therefore we needed to transform the height variable with scaling. We modeled growth at treeline and tundra separately for each year, with host species and inoculation treatment as predictors and the scaled growth metric as the response. We followed the steps described above when testing for an interaction between factors and conducting post hoc tests.

To test whether seedling performance among species varied by inoculation treatment at Arctic treeline and tundra we used linear models. We constrained our analysis to seedlings harvested in tundra in year two due to the low number of seedlings that survived at treeline. We tested whether foliar percentage N and C, Δ^15^N, and Δ ^13^C varied by species, inoculation treatment, and the interaction between the two factors. We calculated Δ^15^N and Δ ^13^C by subtracting the average foliar δ^15^N and δ^13^C signatures for each species at the time inoculation from the foliar signature of each seedling at the time of harvest two years after outplanting. We evaluated specific contrasts of the response of each host species to the mycorrhizal inoculation treatment and across species receiving the same inoculation treatment with a post hoc Tukey p-value adjustment.

We assessed point measurements of the C costs (respiration), C gains (maximum photosynthesis), and CUE associated with each host plant species and inoculation treatment. We tested whether respiration, maximum photosynthesis, and carbon use efficiency varied by host plant species, mycorrhizal inoculation treatment, and the interaction between the two factors.

#### Mycorrhizal colonization

We tested whether mycorrhizal colonization influenced the foliar δ^15^N and δ^13^C of each seedling species two years after outplanting in tundra using linear models. We also separately tested whether mycorrhizal colonization influenced the respiration, maximum photosynthesis, and CUE for each seedling species two years after outplanting in tundra using linear models.

#### Mycorrhizal composition and taxon-specific relative abundance

To assess variation in fungal composition of seedlings among mycorrhizal inoculation treatments, we used nonmetric multidimensional scaling ordinations [51] with the vegan package [52]. We constrained our analysis to seedlings harvested at the tundra site due to low survivorship at the treeline site. The fungal composition of each seedling in the OTU matrix was relativized by total number of OTUs per seedling and transformed using the Beal’s smoothing function to relieve zero truncation [53]. We used the Bray Curtis distance matrix and 500 iterations. We evaluated correlations between specific RAF taxa and variation in RAF composition. We also tested for correlations in RAF composition by host plant species and mycorrhizal inoculation treatment one year and two years after outplanting with the envfit function in the vegan package and conducted post hoc pairwise tests with the RVAideMemoire package [54]. We used indicator species analysis to test whether the relative abundance of each OTU indicated a significant association with host plant species, mycorrhizal inoculation treatment, or duration of outplanting (year one or two) with the indicspecies package (De Caceres & Legendre, 2009). To test whether the fungal communities were more variable in one inoculation treatment than another, by host species, or by year of harvest we assessed multivariate homogeneity of group dispersions with the betadisper function in the vegan package.

We separately ordinated the RAF composition associated with the subset of seedlings harvested two years after outplanting in tundra to see if any of the seedling metrics (foliar traits, gas exchange, growth, biomass) correlated with RAF composition following the same methodology described above. Lastly, we also evaluated Spearman correlations between the relative abundance of the RAF taxa and the foliar δ^15^N and δ^13^C and growth of seedling species outplanted in tundra.

## Results

### Inoculation experiment

**Fig 1:**
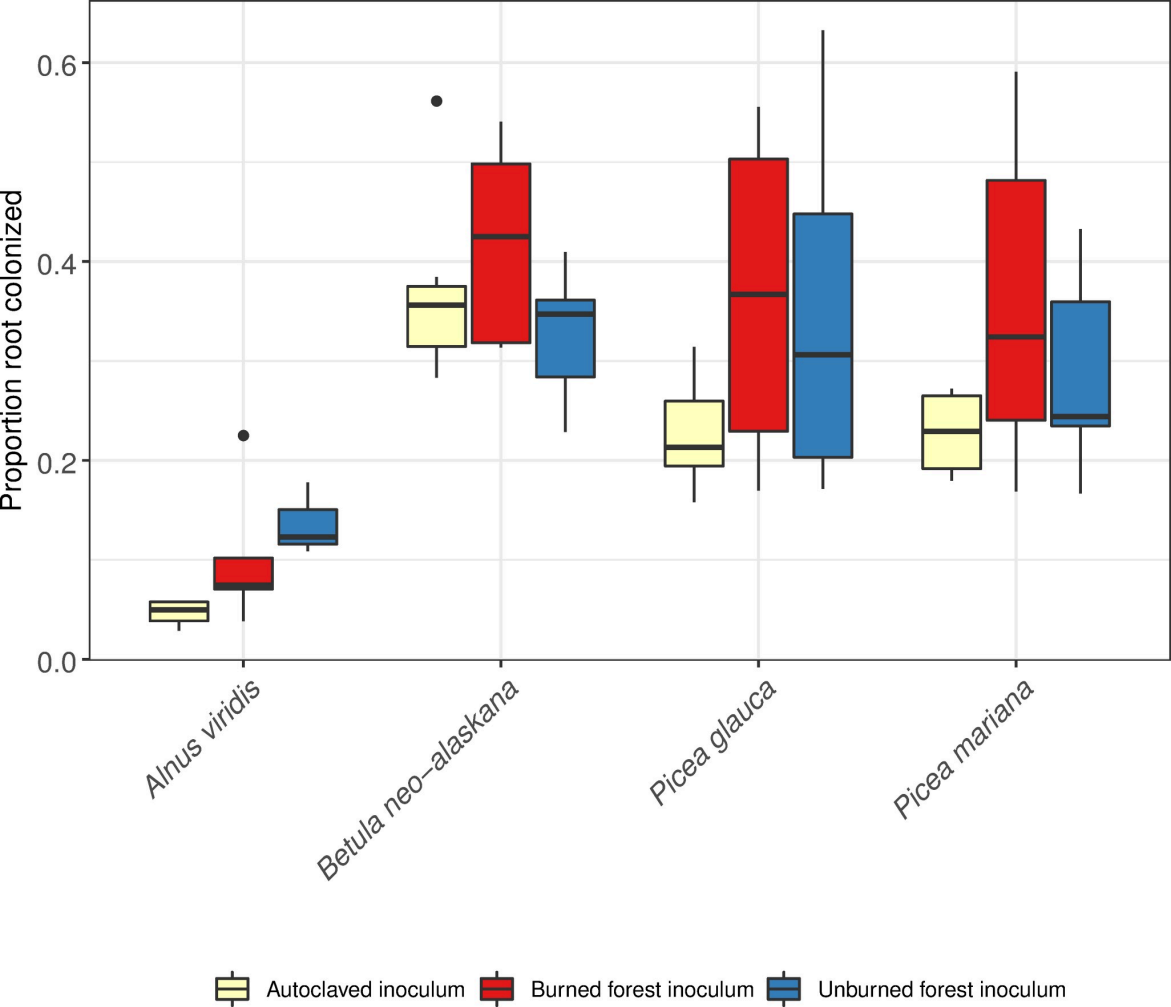
Proportion of fine root length colonized by mycorrhizal fungi for each host plant species and mycorrhizal inoculation treatment. Seedlings were harvested two years after outplanting in Arctic tundra. The lower and upper bounds of the boxplot show the first and third quartiles (the 25^th^ and 75^th^ percentiles), the middle line shows the median, whiskers above and below the boxplot indicate 1.5* inter-quartile range, and points beyond the whiskers indicate outlying points.

Overall, the experimental inoculation treatment increased the proportion of root length colonized by RAF, a strong indication that the inoculation treatment worked. However, the inoculation treatment had no effect on measured seedling responses with the exception of foliar isotope signatures. The proportion of fine root length colonized by mycorrhizal fungi ranged from 3 to 98 percent across all seedlings. The proportion of the root length colonized varied by seedling species (F = 18.54, p = <0.0001) and inoculation treatment (F = 4.81, p = 0.01) but not by the interaction between the two predictors. In general, seedlings with the control inoculation treatment of autoclaved sterile inoculum had the lowest colonization levels (Fig 1). For *A*. *viridis*, *B*. *neo-alaskana*, *P*. *glauca*, and *P*. *mariana*, the autoclaved sterile inoculum treatment had marginally lower colonization than the burned inoculum treatment (p<0.08), but seedlings treated with the burned and unburned forest inoculum had similar colonization levels. Of the species inoculated with the control, burned, or unburned inoculum, *A*. *viridis* had the lowest colonization (p<0.0001) and the other host species did not differ from one another within inoculum treatments.

Seedling survivorship was modest at treeline ranging from 0 to 46% in year one and 0 to 38% in year two across seedling species regardless of inoculation treatment (S1 Table). There was no variation in survivorship of seedling species related to mycorrhizal inoculation treatment at treeline one or two years after outplanting (S2 Table). One year after outplanting *B*. *neo-alaskana* (z-ratio = 2.86, p-value = 0.02) and *P*. *glauca* (z-ratio = 3.00, p-value = 0.01) both had higher survivorship than *P*. *mariana*; yet, these contrasts were not as pronounced by year two. In Arctic tundra survivorship ranged from 83 to 100% for each species in year one and 54 to 100% in year two. Seedling species survivorship again did not respond differently to the mycorrhizal inoculation treatments both one year and two years after outplanting (S2 Table), and the second year after outplanting *P*. *glauca* had higher survivorship than *A*. *viridis* (z-ratio = 2.72, p-value = 0.03), which was not evident in the first year after outplanting.

Seedling growth of each host species responded similarly to all inoculum treatments at treeline but differed among plant species (S3 Table). Overall, *P*. *glauca* had the greatest growth of the seedlings and had significantly greater growth than *B*. *neo-alaskana* (year one T-ratio = 4.78, p-value<0.001, year two T-ratio = 2.97, p-value = 0.02) but similar growth to its congeneric *P*. *mariana*. Seedlings outplanted in the tundra followed the same overall growth relationships to those at treeline with little sensitivity to the inoculation treatment but differences between host species (S3 Table). In tundra, *P*. *glauca* had the greatest growth, greater than *B*. *neo-alaskana* (T-ratio = 4.11, p-value<0.001) and *A*. *viridis* (T-ratio = 4.46, p-value<0.0001) and marginally more than *P*. *mariana* (T-ratio = 2.52, p-value = 0.06), which also had greater growth than *A*. *viridis* (T-ratio = 3.69, p-value<0.01). Growth among species and inoculum treatments was similar in year two in tundra (S3 Table).

Foliar traits were only measured on tundra seedlings two years after outplanting. Foliar percentage N of seedlings was similar among inoculum treatments (F = 0.75, p = 0.47) but varied by host species (F = 25.46, p<0.0001). *Alnus viridis* and *B*. *neo-alaskana* had the highest foliar percentage N compared to both *Picea* species, and *P*. *mariana* had greater foliar N than *P*. *glauca* (S4 Table).

**Fig 2:**
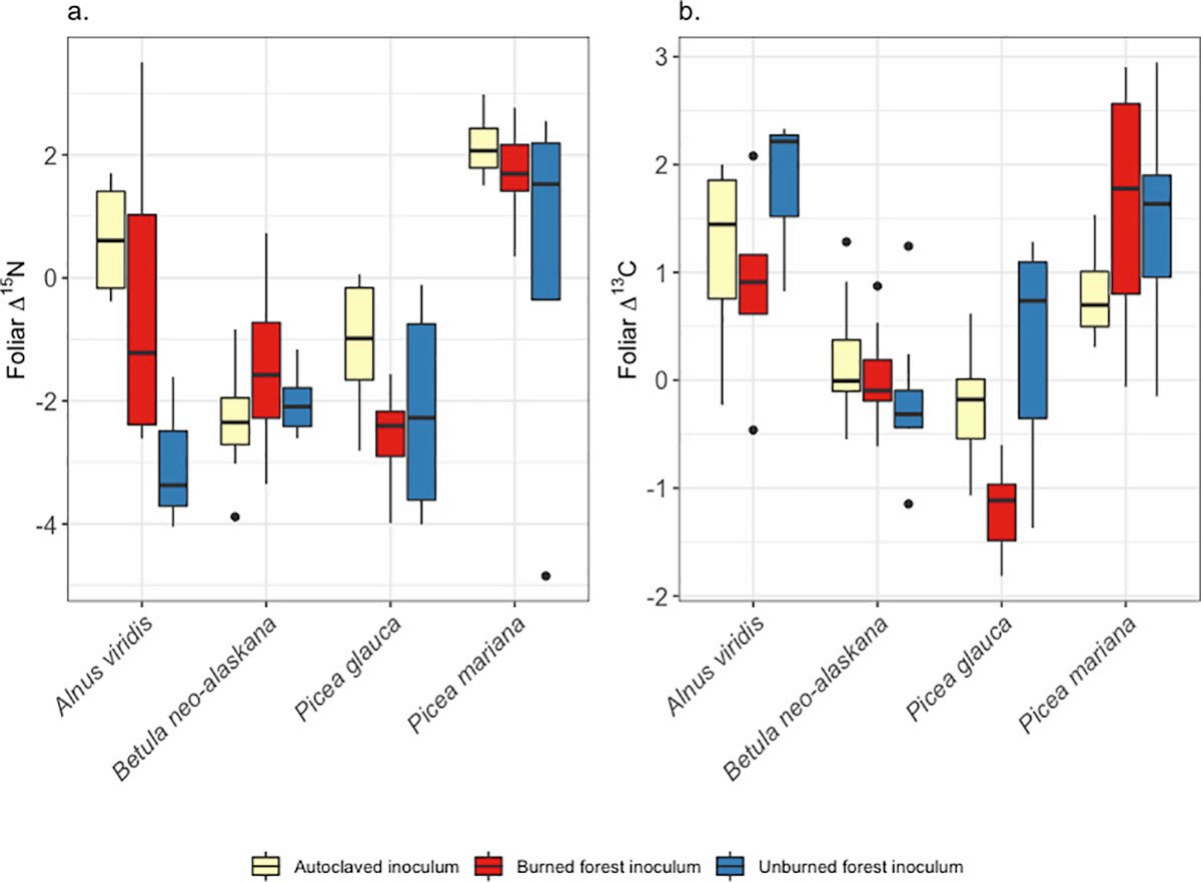
Shifts in isotope signatures of foliar a. nitrogen and b. carbon for seedlings treated with mycorrhizal inoculum and harvested two years after outplanting in Arctic tundra. Δ^15^N and Δ ^13^C were calculated by subtracting the average foliar δ^15^N and δ^13^C for each seedling species at the time inoculation from the foliar signature of each seedling at the time of harvest two years after outplanting. See S6 Table for specific contrasts. The lower and upper bounds of the boxplot show the first and third quartiles (the 25^th^ and 75^th^ percentiles), the middle line shows the median, whiskers above and below the boxplot indicate 1.5* inter-quartile range, and points beyond the whiskers indicate outlying points.

In tundra, both the foliar Δ^15^N and Δ ^13^C of each seedling species differed in their response to the mycorrhizal inoculation treatment (S5 Table). The Δ^15^N ranged from -5.6 to 3.5 ‰ and Δ^13^C from -2.5 to 4.1 ‰ relative to the initial values at outplanting. For *A*. *viridis*, inoculation with the unburned forest inoculum resulted in a depletion of the ^15^N signature (Fig 2A); there were also species level differences within a given inoculum treatment (S6 Table). There was little variation in Δ ^13^C within a species across inoculation treatments, but within an inoculum treatment host species varied in their foliar Δ ^13^C (Fig 2B, S6 Table).

### Mycorrhizal colonization

**Fig 3:**
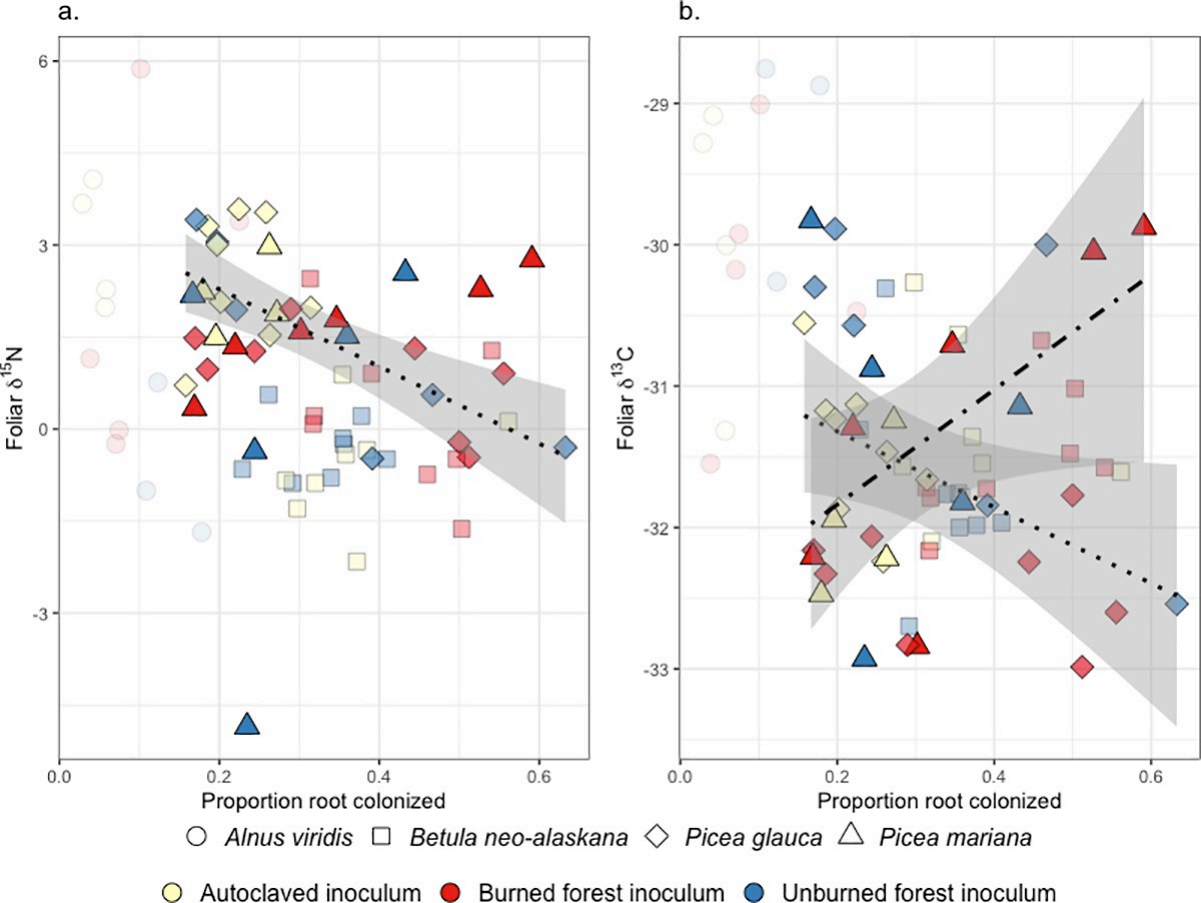
The proportion of root length colonized by root-associated fungi and foliar a. δ^15^N and b. δ^13^C of *Picea glauca* and *Picea mariana*. Panel a regression line fit for *Picea glauca* with S.E. shaded. Panel b regression line fit for *Picea glauca* (dotted) and *Picea mariana* (dot-dashed) with S.E. shaded.

In tundra, foliar δ^15^N ranged from -4.85 to 5.88 and δ^13^C values ranged from -33.82 to -27.76. As RAF colonization increased, the foliar δ^15^N (F = 18.21, p<0.0001, adj R^2^ = 0.45) and δ^13^C declined for *P*. *glauca* (F = 4.56, p = 0.05, adj R^2^ = 0.15) and the foliar δ^13^C marginally increased for *P*. *mariana* (F = 4.66, p = 0.05, adj R^2^ = 0.21, Fig 3)

In tundra, one year after outplanting, the CUE, photosynthesis, and respiration of seedling species responded similarly to the mycorrhizal inoculation treatments, but species varied in these characteristics (S7 Table). By the second year after outplanting, these patterns persisted, but only respiration varied by species (S7 Table). At year one, the deciduous species had higher CUE: *A*. *viridis* had a marginally greater CUE than *P*. *glauca* (T = 2.59, p-value = 0.06) and *B*. *neo-alaskana* (T = 4.18, p-value<0.001). Deciduous species also had higher maximum photosynthesis than the conifers (*A*. *viridis* vs *P*. *glauca* T = 6.04, p-value<0.0001 and *P*. *mariana* T = 3.38, p-value = 0.01; *B*. *neo-alaskana* vs. *P*. *glauca* T = 6.32, p-value<0.0001 and *P*. *mariana* T = 3.54, p-value<0.01). *Picea glauca* had greater respiration than *A*. *viridis* (T = 3.30, p-value = 0.01) in year one, while in year two *B*. *neo-alaskana* had higher respiration than *A*. *viridis* (T = 2.65, p-value = 0.05) and *P*. *mariana* (T = 3.06, p-value = 0.02). In tundra, point measurements of the maximum photosynthesis, respiration, and CUE were not related to proportion of root length colonized (p-value>0.05) for any host species.

### Mycorrhizal composition and taxon-specific relative abundance

**Fig 4:**
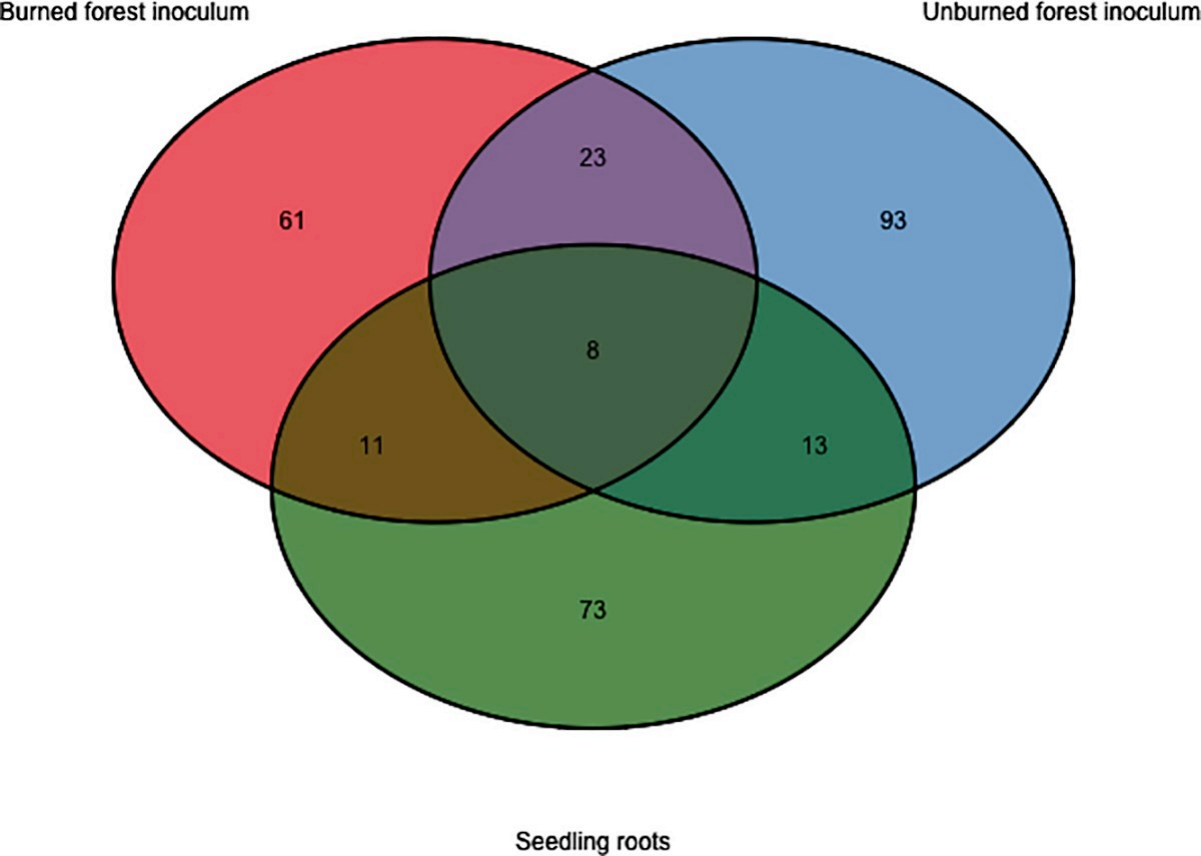
Venn diagram of fungal taxa observed in the live inoculum and on seedling roots.

Our sequencing of the fungal composition of inoculum and of seedling root tips yielded 188 OTUs. These OTUs belonged to the phyla Ascomycota (n = 88), Basidiomycota (n = 97), Mortierellomycota (n = 1) and two taxa were unidentified at the phylum level. The majority of taxa in the Ascomycota were in the order Helotiales and in the Basidiomycota in the Thelephorales, Agaricales, and Russulales. The most abundant taxa in the inoculum and associated with seedling roots were ectomycorrhizal or root endophytes, although saprotrophs and pathogens were also identified. Approximately a quarter of the taxa observed in the burned inoculum were shared with the unburned forest inoculum, and from each inoculum type around a quarter of the taxa were observed on the seedling root systems (Fig 4). Many of these shared taxa were the most abundant taxa observed (S8 Table).

The unburned forest inoculum had 93 OTUs, of which the most abundant were *Russula vinososordida*, *Phialocephala fortinii*, *Cadophora finlandica*, *Hymenoscyphus epiphyllus*, *Hebeloma bruchetii*, *Alnicola* sp, *Wilcoxina rehmii* and several other *Russula* sp. The burned forest inoculum had 61 OTUs of which the most abundant were *C*. *finlandica*, *Thelephora terrestris*, *Ramaria conjunctipes*, *Lactarius pubescens*, and *Meliniomyces bicolor*. Thirty-nine of the 73 OTUs observed on the root systems of outplanted seedlings were identified as RAF, in this case either ectomycorrhizal fungi or root endophytes. The most dominant RAF were identified as *T*. *terrestris*, *M*. *variabilis*, *P*. *fortinii*, and *C*. *finlandica*, many of which were also the dominant taxa across the two experimental inoculum sources.

**Fig 5:**
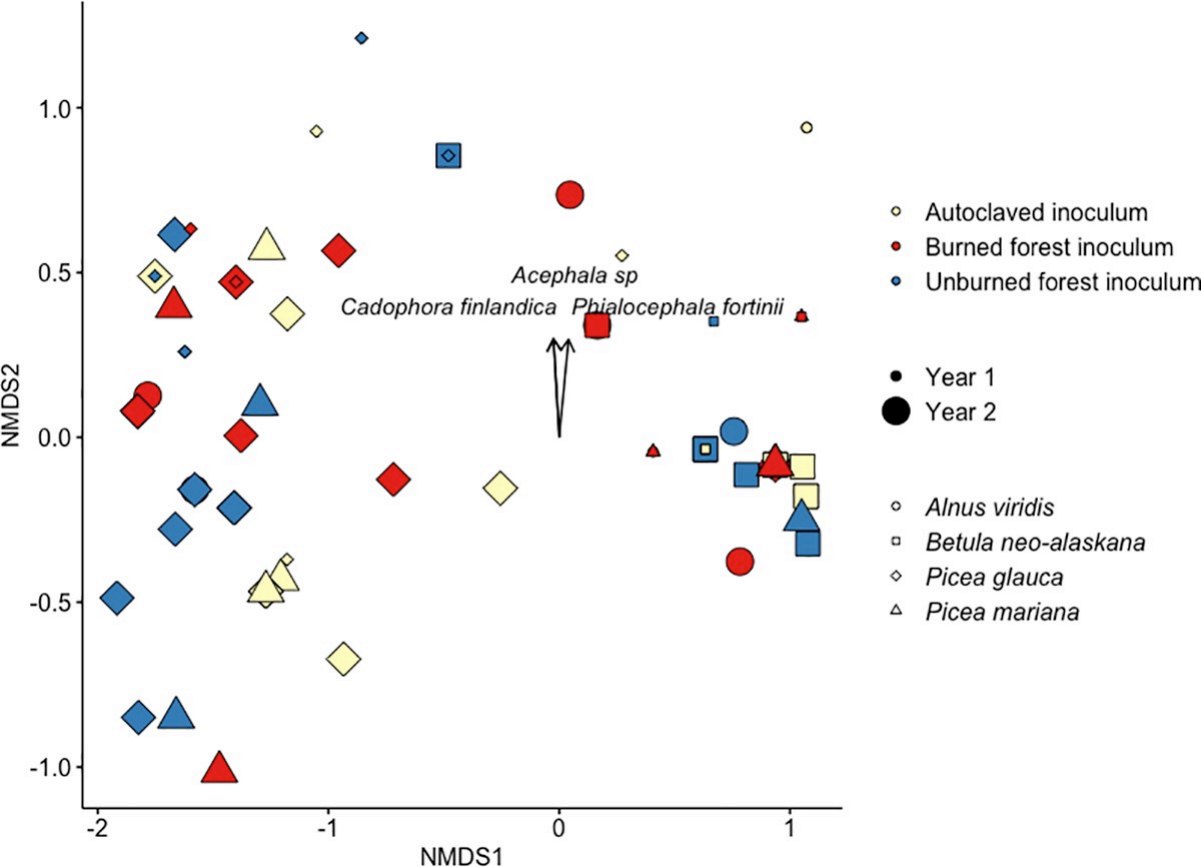
NMDS bi-plot of root-associated fungi (RAF) associated with seedlings outplanted for one or two years in Arctic tundra. Vectors show direction and magnitude of significant correlations between RAF composition and RAF taxa.

The final ordination of seedling RAF composition had three dimensions, a stress of 0.04 indicating a good fit, a non-metric fit R^2^ = 0.998, and linear fit R^2^ = 0.993 (Fig 5). Four taxa were strongly associated with variation in RAF composition across seedlings: *P*. *fortinii* (r^2^ = 0.06, p = 0.05), two taxa identified as *C*. *finlandica* (= 0.09, p = 0.01; r^2^ = 0.15, p = 0.02) and *Acephala* sp. (r^2^ = 0.09, p = 0.01).

Surprisingly, root-associated fungal composition did not vary with inoculum treatment (r^2^<0.01, p = 0.95), but instead was related to host plant species (r^2^ = 0.50, p = 0.001) and duration of outplanting (r^2^ = 0.03, p = 0.04). There was no difference in homogeneity of variance associated with inoculum treatment (F = 0.07, p = 0.93) or year of harvest (F = 0.01, p = 0.92) but there was associated with seedling species (F = 22.56, p<0.0001). *Betula neo-alaskana* had less dispersion than *A*. *viridis* but greater variance than both spruce species (p<0.0001). One DSE taxon, *Sistotrema alboluteum* (IndVal = 0.384, p = 0.004), was an indicator taxon of the burned inoculum and an EMF taxon, *Tomentella ellisii* (IndVal = 0.285, p = 0.026), was an indicator taxon of unburned forest inoculum. The RAF composition associated with *A*. *viridis* and *P*. *mariana* differed from *B*. *neo-alaskana* and *P*. *glauca* (p<0.05), yet these two did not have different composition from one another. There were two fungal taxa that were strongly associated with RAF community composition one year after outplanting, both with the closest RDP assignment to *T*. *terrestris* (IndVal = 0.51, p<0.01 and IndVal = 0.31, p = 0.02). The abundance of *P*. *fortinii* was strongly associated with variation in RAF composition two years after outplanting (IndVal = 0.43, p = 0.03). There were two indicator taxa of *A*. *viridis*, a taxon identified as *Alnicola* sp. (IndVal = 0.485, p< 0.01) and *T*. *terrestris* (IndVal = 0.40, p = 0.02). There were two indicator taxa of *P*. *glauca*, a taxon identified as *C*. *finlandica* (IndVal = 0.485, p< 0.01) and the endophyte *Varicosporium elodeae* (IndVal = 0.40, p = 0.02). When host species were grouped we saw that *P*. *fortinii* (IndVal = 0.57, p<0.001) was a significant indicator of *A*. *viridis* and *P*. *glauca*, while two taxa identified as *T*. *terrestris* (IndVal = 0.84, p<0.001; IndVal = 0.49, p = 0.05) were indicators of *A*. *viridis*, *B*. *neo-alaskana*, and *P*. *mariana*, and *M*. *variabilis* (IndVal = 0.57, p<0.01) was associated with the group of host plants including *A*. *viridis*, *P*. *glauca*, and *P*. *mariana*.

**Fig 6:**
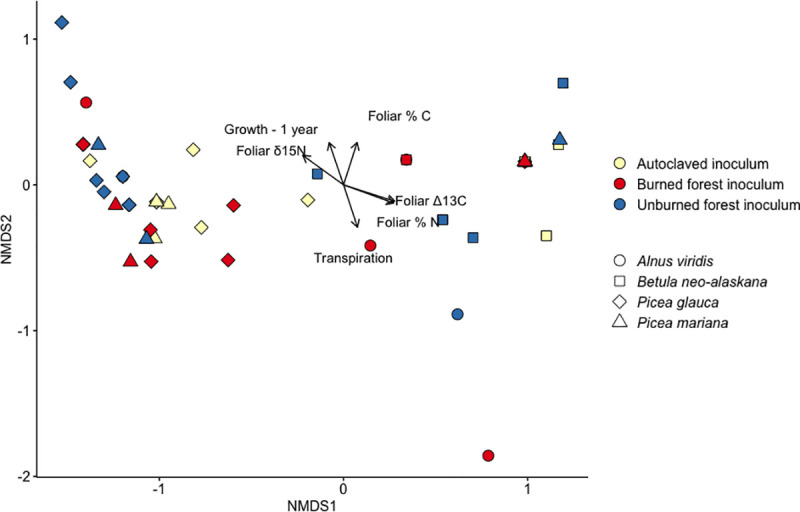
NMDS bi-plot of root-associated fungi (RAF) associated with seedlings outplanted for two years in Arctic tundra. Vectors show direction and magnitude of significant correlations between RAF composition and seedling metrics.

The final ordination of the RAF composition associated with seedlings harvested two years after outplanting in tundra had three dimensions, a stress of 0.03 indicating an excellent fit, a non-metric fit R^2^ = 0.999, and linear fit R^2^ = 0.996 (Fig 6). Again RAF composition did not vary with inoculation treatment (r^2^ = 0.01, p = 0.51) and instead was related to host plant species (r^2^ = 0.48 p = 0.001). Fungal composition was related to growth between inoculation and the first year of harvest, foliar N and C percentage, foliar δ^15^N, foliar Δ13C, and transpiration measured during the gas exchange measurements (p<0.05, Fig 6, S9 Table)

**Fig 7:**
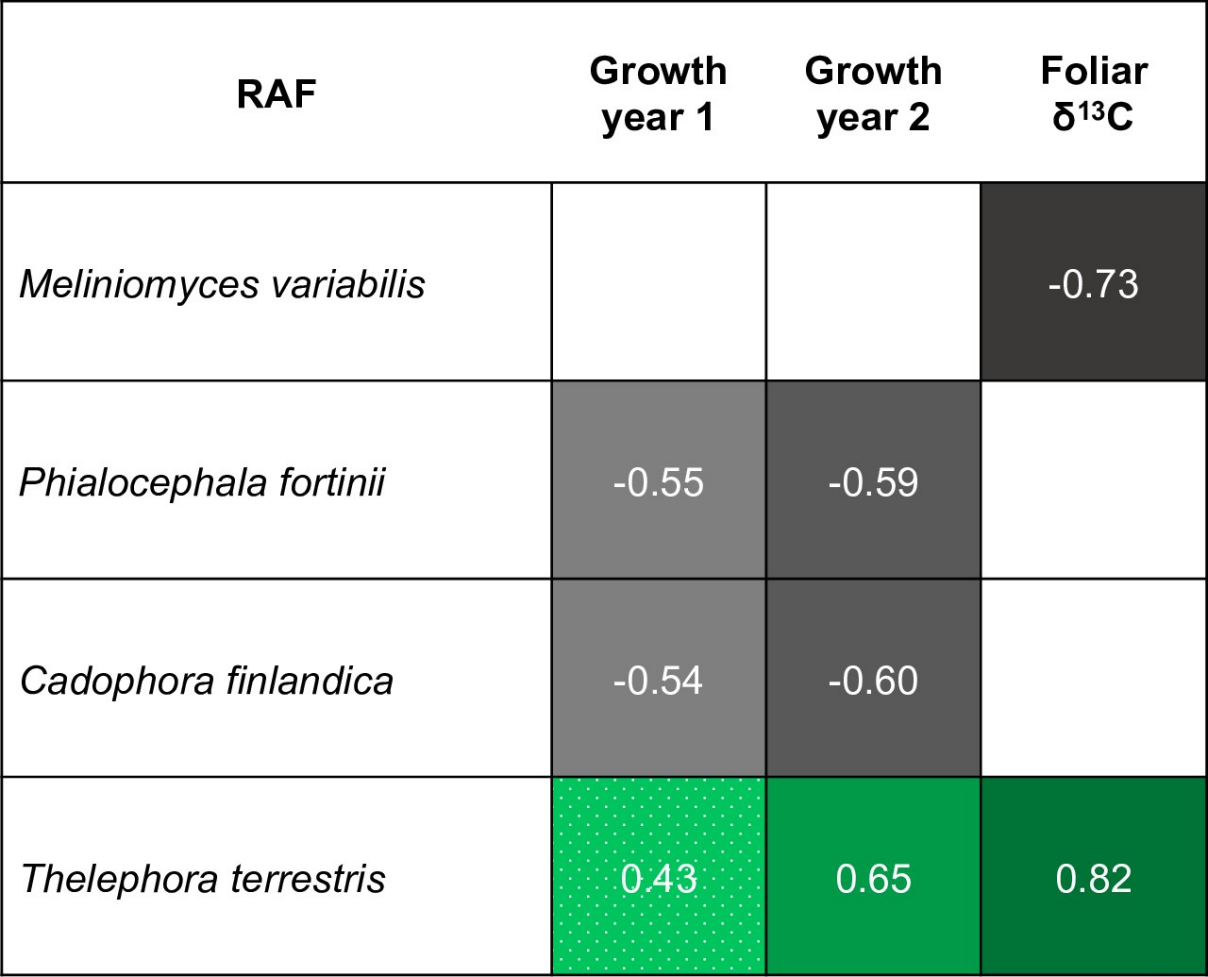
Spearman correlations between the traits of seedlings outplanted in tundra and harvest after two years and the relative abundance of root-associated fungi. Green colors indicate positive and gray indicates negative Spearman Rho correlation coefficients. All relationships are significant at p<0.05. Solid colors indicate correlations with *Picea mariana* and the dotted pattern indicates correlation with *Betula neo-alaskana*.

There were notable correlations between the relative abundance of RAF taxa and the physiological performance of seedlings outplanted in tundra (Fig 7). The growth of *B*. *neo-alaskana*, and *P*. *mariana* had significant correlations with particular RAF taxa. The endophytes *C*. *finlandica* (closest ID to SH027361.07FU_AF486119) and *P*. *fortinii* (closest ID to SH020813.07FU_AY033087) were negatively correlated with growth of *P*. *mariana* in year one and two. Conversely, the growth of *B*. *neo-alaskana* in year one and *P*. *mariana* in year two was greater for seedlings with higher relative abundance of the ectomycorrhizal taxon, *T*. *terrestris* (closest ID to SH006801.07FU_JX990010). Following this pattern of fungal effects on plant performance by guild, the relative abundance of the endophyte *M*. *variabilis* (closest ID to SH004575.07FU_AY762619) was negatively correlated the foliar δ^13^C of *P*. *mariana*, indicating that associations with the endophyte were correlated with enhanced photosynethic capacity; whereas, the relative abundance of the ectomycorrhizal taxon, *T*. *terrestris* was positively associated with δ^13^C of *P*. *mariana*, indicating photosynthetic capacity was reduced with greater fungal abundance. *Cadophora finlandica*, *P*. *fortinii*, and *T*. *terrestris* occurred in both the burned and unburned forest inoculum and on the root systems of outplanted seedlings. *Phialocephala fortinii* and *T*. *terrestris* were also observed on the root systems of seedlings that received the autoclaved inoculum suggesting that these OTUs were also resident taxa of the outplant site. *Meliniomyces variabilis* was in the burned forest inoculum but also observed on seedling that received the burned forest, unburned forest and autoclaved inoculum treatments again suggesting colonization from local fungi. Together this suggests that resident inoculum from the outplant sites colonized roots and had strong physiological impacts. Endophytes that associate with ERM hosts or belong to the DSE guild had apparent negative or neutral effects on plant performance, while one ectomycorrhizal taxon showed a positive effect on plant growth but may have reduced photosynthetic capacity.

## Discussion

The controls over seedling establishment are a major source of uncertainty in predicting future vegetation transitions in the Arctic, particularly after wildfire. We sought to explore whether the establishment of boreal forest seedlings and shrubs at and beyond current treeline was limited by a lack of mycorrhizal symbionts. Our study did not find support for mycorrhizal limitation on seedling establishment given there was no effect of mycorrhizal inoculation treatment on seedling survival and growth. Instead we observed that tree and shrub seedlings responded similarly to a sterile, autoclaved control inoculation and the provision of fire-affected or unburned boreal forest inoculum. Seedlings appeared to be colonized by resident fungi, which likely diluted the potential inoculation effect, and suggests that compatible fungi are present at and beyond the range limit of the studied host plant species. Even though the recruitment of seedlings was not related to the inoculum treatment *per se*, we found strong evidence that performance was related to the composition of the RAF community and the relative abundance of particular RAF. Foliar isotope signatures, often used as indicators of plant nutrient status and photosynthetic capacity, and the shift in these signatures over time (Δ^15^N and Δ ^13^C) were responsive to the inoculum treatment, RAF community composition, RAF colonization, and in some cases the relative abundance of particular RAF taxa. Together our findings suggest that 1) seedling establishment may be supported by plant-fungal associations of resident post-fire RAF communities as much as fungal communities from burned and unburned boreal forest, 2) co-migration of boreal fungi is not necessary for seedling establishment after fires at and beyond treeline, and 3) the identities of mycorrhizal fungi are important to post-establishment seedling performance under field conditions.

Boreal seedling survival and growth responded similarly to the provision of RAF communities from burned and unburned boreal forest inoculum and the autoclaved control inoculum treatment. This could mean that seedling establishment is not affected by mycorrhizal associations in the early phases of establishment. In heathlands ectomycorrhizal seedlings have been shown to survive at least one year waiting for appropriate ectomycorrhizal symbionts after establishment in ericoid dominated vegetation [55], which is similar to our observations in tundra. Alternatively, the fungi provided in the inoculum treatments may have equivalent impacts on seedling establishment as the resident fungi from the outplant sites. The fungi observed associating with seedlings that received the autoclaved inoculum treatment were some of the most abundant in the sequence datasets: *T*. *terrestris*, *M*. *variabilis*, *P*. *fortinii*, *V*. *elodeae*, and *R*. *ericae*. These fungi are known to have fairly wide distributions, but for many their ecological impacts are less well understood. *Thelephora terrestris* can reduce root pathogen infection [11] and pathogen abundance can increase with fire severity [30], suggesting a beneficial role of *T*. *terrestris* post-fire. However, *T*. *terrestris* is likely a species complex [56], which includes at least one alder-specific lineage [57]. Thus, the *T*. *terrestris* OTUs colonizing our host species may not be functionally redundant. The other RAF observed to colonize control seedlings are known DSE fungi or symbionts of ericaceous plants. We observed a negative effect of DSE taxa on seedling growth and a neutral growth response to the ERM taxon similar to observations from interior boreal forest sites [36]. Little is known about the importance of these fungi to plant performance in field conditions or how variation in their abundances may co-vary with ectomycorrhizal seedling establishment [33, 58–60]. Meta-analysis of the effects of endophyte colonization under controlled experimental conditions indicates a neutral or negative effect of DSE on plant growth, particularly for *Picea* sp. and hosts colonized by *P*. *fortinii* [61].

Notably, recipients of the burned or unburned forest inoculum showed greater variation in some of the seedling traits like proportion of roots colonized and foliar isotope signatures. There may be a stochastic component to which fungi survived and colonized a seedling with our inoculation technique. The observed fungal composition suggests there is a fairly generic, universal inoculum available in the outplant sites, but our inoculation may have occasionally provided distinctly different fungi, increasing the variance in response variables such as proportion of root colonized or foliar isotopes. Surprisingly, fungal composition and the variance within communities did not differ by mycorrhizal inoculum treatment.

RAF composition and the variance in community structure varied with seedling species. This suggests that the RAF that ultimately colonized roots may be selected for by host plants from the greater species pool of RAF in the inoculum treatments as well as those present in the resident post-fire community. With the exception of alder, this was unexpected because the tree seedling species we outplanted have all been shown to associate with generalist fungal communities [36, 62]. Even *A*. *viridis*, which is known to associate with a narrower suite of fungi, can share taxa with other boreal ectomycorrhizal hosts [36, 63]. Our indicator species analysis revealed that fairly common fungal associates like *T*. *terrestris*, *M*. *variabilis*, and *P*. *fortinii*, varied in their abundance based on host plant, resulting in potentially strong differences in physiological performance related to seedling carbon dynamics and growth. In previous research at the treeline site, we observed strong correlations between seedling RAF composition and the foliar traits and biomass of naturally established seedlings [15]. We can speculate that depending on the time frame over which seedlings select particular fungi, RAF may have influenced seedling establishment given that survival and growth varied by host plant in the current study. This, however, cannot be confirmed with this dataset because we don’t know what fungi were associated with seedlings that did not survive.

In general, there was modest survivorship of outplanted seedlings at treeline and fairly high survivorship in tundra. We infer that strong abiotic effects may have driven these difference. Surprisingly, our point measurements of soil moisture at the treeline site were similar to the tundra site (VWC FM 34.1 ±2.9 S.E., ARF 35.5 ±1.07S.E), suggesting that at least from our snapshot measurements, soil moisture alone was not an important driver of survival. The strongest abiotic driver may have been related to wind damage. We observed apical death and in some cases resprouting of many seedlings at the treeline site. Wind damage and desiccation is an important determinant of tree seedling success at other treeline locations [64, 65].

*Picea glauca*, the coniferous species most often associated with Alaska treeline, had the highest survival and also consistently had the greatest growth at treeline and in tundra. Despite, *P*. *glauca*’s lack of sensitivity to the mycorrhizal inoculation treatments, this host plant had the strongest relationship with other fungal parameters like RAF colonization levels. On broader geographic scales, mycorrhizal colonization of mature *P*. *glauca* was correlated with tree growth [66]. This suggests that the effects of plant-fungal interactions on *P*. *glauca* performance that are apparent at the seedling phase may persist as *P*. *glauca* ages.

Seedling performance was correlated with RAF colonization of fine root length. We observed inverse relationship between foliar δ^15^N of *P*. *glauca* with colonization, consistent with higher reliance on mycorrhizal fungi for sourcing N. This supports findings from a greenhouse study where there was a depletion of foliar δ^15^N with the colonization of *Pinus sylvestris* fine roots by mycorrhizal fungi [67] and an increase in foliar δ^13^C, but only under low N conditions [68]. We observed that as seedlings supported a higher proportion of root length with RAF, *P*. *mariana* and *P*. *glauca* showed contrasting responses in their foliar δ^13^C. δ^13^C was more enriched with higher RAF colonization for *P*. *mariana*, and in particular *T*. *terrestris* abundance, which may indicate greater drought stress or constraint on photosynthesis as allocation to RAF increases; whereas the foliar δ^13^C was more depleted for *P*. *glauca* with higher colonization, which indicates relatively lower drought stress or less constraint on photosynthesis as allocation to RAF increases. In general across species δ^13^C was positively related to percentage N, which suggests that wind or drought stress more than photosynthetic capacity may be the mechanism that influenced isotope signatures.

The composition of unburned and burned inoculum supports the conceptual framework of ‘late-stage’ fungi being present in mature forests and ‘early-stage’ fungi being abundant after disturbance [26, 27, 69]. We observed ‘late-stage’ ECM fungi in the forest inoculum, but not in the burned inoculum and never on the seedlings. At the treeline location, in previous research, we did observe that establishing seedlings could associate with ‘late-stage’ fungi when the seedlings established in close proximity to a host plant that survived fire and resprouted [15]. In that study, we speculated that seedlings were tapping into a common mycorrhizal network. We would expect that supporting these ‘late-stage’ fungi would have greater carbon costs, which in this study would not be offset by seedling integration into a common mycorrhizal network, thus preventing these associations from persisting.

## Conclusions

To our knowledge this is the first study to experimentally test whether the provision of mycorrhizal inoculum facilitates tree and shrub establishment under field conditions and thus vegetation transitions in the Arctic after fire. We observed that boreal tree and shrub seedling survival was not affected by the experimental provision of boreal forest fungal inoculum designed to mimic co-migration. Instead, post-establishment performance was affected by the RAF community composition, colonization and the relative abundance of particular RAF taxa that included many resident treeline and tundra fungi. *Picea glauca* had the highest survivorship and growth at and beyond current treeline and its foliar traits were also most sensitive to mycorrhizal colonization. We show that *P*. *glauca*, the resident treeline species, can successfully establish at and beyond current treeline and that its post-establishment performance will be affected by the mycorrhizal partners that are available. We also show, however, that vegetation transitions from tundra to forest are not limited by the obligate co-migration of boreal forest fungi as tree seedlings disperse at and beyond their range limit, at least within the first two years of establishment. We illuminate the functional redundancy of unburned forest, burned forest and burned tundra fungal composition and the potential for widely-distributed, abundant mycorrhizal and endophytic taxa to support seedling establishment.

## Supporting information

S1 Table Percentage survived for each host species outplanted at Arctic treeline and tundra one and two years after outplanting. Survivorship was not influenced by mycorrhizal inoculation treatment.
(DOCX)

S2 Table Analysis of Deviance from logistic regression testing the effect of mycorrhizal inoculation treatment on seedling survivorship of four host plant species. (DOCX)

S3 Table The effect of mycorrhizal inoculation treatment on seedling growth of four host plant species outplanted in Arctic tundra and treeline modeled with linear regression. (DOCX)

S4 Table Contrasts of foliar percentage N of host plant species outplanted in Arctic tundra. Foliar percentage N did not differ with mycorrhizal inoculation treatment.
(DOCX)

S5 Table The effect of mycorrhizal inoculation treatment and host species on the shift in foliar isotope signatures (Δ^15^N and Δ ^13^C) between the time of outplanting and harvest two years after outplanting in Arctic tundra. (DOCX)

S6 Table Specific contrasts between species receiving the autoclaved control (S), burned (B) or unburned (F) forest inoculum treatment. A- *Alnus viridis*, B—*Betula neo-alaskana*, G-*Picea glauca*, M- *Picea mariana*. Bolded estimates indicate significant contrasts with Tukey p-value adjustment.
(DOCX)

S7 Table The effect of mycorrhizal inoculation treatment and host species on carbon use efficiency, photosynthesis, and respiration measured on seedlings outplanted in Arctic tundra. (DOCX)

S8 Table Fungal operational taxonomic units observed in burned and unburned boreal forest inoculum and on the root systems of seedlings outplanted in Arctic tundra. (XLSX)

S9 Table Correlations between seedling traits and RAF composition of seedlings harvested two years after outplanting in Arctic tundra. Bold indicates significant variables.
(DOCX)
