# Supplementary material for: Limited overall impacts of ectomycorrhizal inoculation on recruitment of boreal trees into Arctic tundra following wildfire belie species-specific responses
Source: PLoS One. 2020 Jul 9;15(7):e0235932. doi: 10.1371/journal.pone.0235932 (PMC7347221; doi:10.1371/journal.pone.0235932)
Supplement: S1 Table — Survivorship was not influenced by mycorrhizal inoculation treatment. (DOCX) [file pone.0235932.s001.docx]

S1a Table. Percentage survived for each host species outplanted at Arctic treeline and tundra one and two years after outplanting. Survivorship was not influenced by mycorrhizal inoculation treatment.

|  | Arctic treeline | | Arctic tundra | |
| --- | --- | --- | --- | --- |
| Species | year one | year two | year one | year two |
| *Alnus viridis* | 0 | 0 | 92 | 54 |
| *Betula neo-alaskana* | 46 | 29 | 100 | 100 |
| *P. glauca* | 42 | 38 | 100 | 96 |
| *P. mariana* | 8 | 17 | 83 | 63 |
