# Supplementary material for: Limited overall impacts of ectomycorrhizal inoculation on recruitment of boreal trees into Arctic tundra following wildfire belie species-specific responses
Source: PLoS One. 2020 Jul 9;15(7):e0235932. doi: 10.1371/journal.pone.0235932 (PMC7347221; doi:10.1371/journal.pone.0235932)
Supplement: S2 Table — (DOCX) [file pone.0235932.s002.docx]

S1b Table. Analysis of Deviance from logistic regression testing the effect of mycorrhizal inoculation treatment on seedling survivorship of four host plant species

|  |  |  | χ^2^ | Df | p-value |
| --- | --- | --- | --- | --- | --- |
| Treeline | Year 1 | Treatment | 1.42 | 2 | 0.49 |
|  |  | Species | 44.88 | 3 | <0.001 |
|  | Year 2 | Treatment | 1.76 | 2 | 0.42 |
|  |  | Species | 16.6 | 3 | <0.001 |
| Tundra | Year 1 | Treatment | 0.59 | 2 | 0.74 |
|  |  | Species | 17.15 | 3 | <0.001 |
|  | Year 2 | Treatment | 1.47 | 2 | 0.48 |
|  |  | Species | 28.02 | 3 | <0.001 |
