# Supplementary material for: Limited overall impacts of ectomycorrhizal inoculation on recruitment of boreal trees into Arctic tundra following wildfire belie species-specific responses
Source: PLoS One. 2020 Jul 9;15(7):e0235932. doi: 10.1371/journal.pone.0235932 (PMC7347221; doi:10.1371/journal.pone.0235932)
Supplement: S3 Table — (DOCX) [file pone.0235932.s003.docx]

S2 Table. The effect of mycorrhizal inoculation treatment on seedling growth of four host plant species outplanted in Arctic tundra and treeline modeled with linear regression.

|  |  |  | F-value | Df | p-value |
| --- | --- | --- | --- | --- | --- |
| Treeline | Year 1 | Treatment | 0.56 | 2 | 0.59 |
|  |  | Species | 11.92 | 3 | <0.001 |
|  | Year 2 | Treatment | 0.65 | 2 | 0.54 |
|  |  | Species | 4.43 | 3 | 0.03 |
| Tundra | Year 1 | Treatment | 0.42 | 2 | 0.66 |
|  |  | Species | 1.46 | 3 | <0.001 |
|  | Year 2 | Treatment | 0.18 | 2 | 0.84 |
|  |  | Species | 2.06 | 3 | 0.11 |
