# Supplementary material for: Limited overall impacts of ectomycorrhizal inoculation on recruitment of boreal trees into Arctic tundra following wildfire belie species-specific responses
Source: PLoS One. 2020 Jul 9;15(7):e0235932. doi: 10.1371/journal.pone.0235932 (PMC7347221; doi:10.1371/journal.pone.0235932)
Supplement: S4 Table — Foliar percentage N did not differ with mycorrhizal inoculation treatment. (DOCX) [file pone.0235932.s004.docx]

S3 Table. Contrasts of foliar percentage N of host plant species outplanted in Arctic tundra. Foliar percentage N did not differ with mycorrhizal inoculation treatment.

| Species contrasts | Estimate ± S.E. | T-ratio | p-value |
| --- | --- | --- | --- |
| *Alnus viridis - Betula neo-alaskana* | -0.07 ± 0.14 | -0.49 | 0.96 |
| *Alnus viridis - Picea glauca* | 0.78 ± 0.14 | 5.7 | <0.0001 |
| *Alnus viridis - Picea mariana* | 0.39 ± 0.15 | 2.63 | 0.05 |
| *Betula neo-alaskana - Picea glauca* | 0.85 ± 0.10 | 8.21 | <0.0001 |
| *Betula neo-alaskana - Picea mariana* | 0.46 ± 0.12 | 3.88 | <0.01 |
| *Picea glauca - Picea mariana* | -0.39 ± 0.12 | -3.31 | <0.01 |
