# Supplementary material for: Limited overall impacts of ectomycorrhizal inoculation on recruitment of boreal trees into Arctic tundra following wildfire belie species-specific responses
Source: PLoS One. 2020 Jul 9;15(7):e0235932. doi: 10.1371/journal.pone.0235932 (PMC7347221; doi:10.1371/journal.pone.0235932)
Supplement: S5 Table — (DOCX) [file pone.0235932.s005.docx]

S4a Table. The effect of mycorrhizal inoculation treatment and host species on the shift in foliar isotope signatures (Δ^15^N and Δ ^13^C) between the time of outplanting and harvest two years after outplanting in Arctic tundra.

|  |  | F-value | Df | p-value |
| --- | --- | --- | --- | --- |
| Δ^15^N | Treatment | 4.14 | 2 | 0.02 |
|  | Species | 21.14 | 3 | <0.001 |
|  | Treatment x species | 2.57 | 6 | 0.03 |
| Δ ^13^C | Treatment | 2.19 | 2 | 0.12 |
|  | Species | 21.96 | 3 | <0.0001 |
|  | Treatment x species | 2.58 | 6 | 0.03 |
