# Supplementary material for: Limited overall impacts of ectomycorrhizal inoculation on recruitment of boreal trees into Arctic tundra following wildfire belie species-specific responses
Source: PLoS One. 2020 Jul 9;15(7):e0235932. doi: 10.1371/journal.pone.0235932 (PMC7347221; doi:10.1371/journal.pone.0235932)
Supplement: S6 Table — A- Alnus viridis, B—Betula neo-alaskana, G-Picea glauca, M- Picea mariana. Bolded estimates indicate significant contrasts with Tukey p-value adjustment. (DOCX) [file pone.0235932.s006.docx]

S4b Table. Specific contrasts between species receiving the autoclaved control (S), burned (B) or unburned (F) forest inoculum treatment. A- *Alnus viridis*, B - *Betula neo-alaskana*, G-*Picea glauca*, M- *Picea mariana*. Bolded estimates indicate significant contrasts with Tukey p-value adjustment.

|  | Δ^15^N | Δ ^13^C |
| --- | --- | --- |
|  | Estimate ± S.E. | Estimate ± S.E. |
| A.S-A.B | 0.97 ± 0.94 | 0.31 ± 0.53 |
| A.S-A.F | **3.64 ± 1.07** | -0.63 ± 0.6 |
| A.B-A.F | 2.67 ± 1.03 | -0.93 ± 0.57 |
| B.S-B.B | -0.87 ± 0.7 | 0.16 ± 0.39 |
| B.S-B.F | -0.31 ± 0.7 | 0.37 ± 0.39 |
| B.B-B.F | 0.57 ± 0.7 | 0.21 ± 0.39 |
| G.S-G.B | 1.56 ± 0.7 | 0.96 ± 0.39 |
| G.S-G.F | 1.1 ± 0.76 | -0.56 ± 0.42 |
| G.B-G.F | -0.46 ± 0.76 | -1.52 ± 0.42 |
| M.S-M.B | 0.46 ± 0.91 | -0.81 ± 0.51 |
| M.S-M.F | 1.94 ± 0.94 | -0.65 ± 0.53 |
| M.B-M.F | 1.48 ± 0.85 | 0.16 ± 0.47 |
| A.S-B.S | **2.97 ± 0.86** | 0.97 ± 0.48 |
| A.S-G.S | 1.69 ± 0.86 | 1.41 ± 0.48 |
| A.S-M.S | -1.52 ± 1 | 0.36 ± 0.55 |
| B.S-M.S | **-4.49 ± 0.86** | -0.62 ± 0.48 |
| B.S-G.S | -1.28 ± 0.7 | 0.44 ± 0.39 |
| M.S-G.S | **3.21 ± 0.86** | 1.05 ± 0.48 |
| A.B-B.B | 1.13 ± 0.8 | 0.83 ± 0.45 |
| A.B-G.B | 2.29 ± 0.8 | 2.06 ± 0.45 |
| A.B-M.B | -2.03 ± 0.85 | -0.76 ± 0.47 |
| B.B-M.B | **-3.16 ± 0.76** | **-1.58 ± 0.42** |
| B.B-G.B | 1.16 ± 0.7 | 1.23 ± 0.39 |
| M.B-G.B | **4.31 ± 0.76** | **2.82 ± 0.42** |
| A.F-B.F | -0.98 ± 0.95 | **1.96 ± 0.53** |
| A.F-G.F | -0.85 ± 1 | 1.48 ± 0.55 |
| A.F-M.F | -3.22 ± 1.03 | 0.33 ± 0.57 |
| B.F-M.F | -2.24 ± 0.8 | **-1.63 ± 0.45** |
| B.F-G.F | 0.13 ± 0.76 | -0.49 ± 0.42 |
| M.F-G.F | 2.37 ± 0.85 | 1.14 ± 0.47 |
