# Supplementary material for: Limited overall impacts of ectomycorrhizal inoculation on recruitment of boreal trees into Arctic tundra following wildfire belie species-specific responses
Source: PLoS One. 2020 Jul 9;15(7):e0235932. doi: 10.1371/journal.pone.0235932 (PMC7347221; doi:10.1371/journal.pone.0235932)
Supplement: S7 Table — (DOCX) [file pone.0235932.s007.docx]

S5 Table. The effect of mycorrhizal inoculation treatment and host species on carbon use efficiency (CUE), photosynthesis, and respiration measured on seedlings outplanted in Arctic tundra.

|  |  | CUE | | | Photosynthesis  (µmol m-2 s-1) | | | Respiration  (µmol m-2 s-1) | | |
| --- | --- | --- | --- | --- | --- | --- | --- | --- | --- | --- |
|  |  | F-value | Df | p-value | F-value | Df | p-value | F-value | Df | p-value |
| year 1 | Treatment | 1.49 | 2 | 0.24 | 0.05 | 2 | 0.95 | 1.1 | 2 | 0.34 |
|  | Species | 5.91 | 3 | <0.01 | 18.49 | 3 | <0.0001 | 4.22 | 3 | 0.01 |
| year 2 | Treatment | 0.63 | 2 | 0.54 | 0.88 | 2 | 0.42 | 0.94 | 2 | 0.4 |
|  | Species | 2.28 | 3 | 0.09 | 1.78 | 3 | 0.16 | 4.47 | 3 | 0<0.01 |
