# Supplementary material for: Limited overall impacts of ectomycorrhizal inoculation on recruitment of boreal trees into Arctic tundra following wildfire belie species-specific responses
Source: PLoS One. 2020 Jul 9;15(7):e0235932. doi: 10.1371/journal.pone.0235932 (PMC7347221; doi:10.1371/journal.pone.0235932)
Supplement: S9 Table — Bold indicates significant variables. (DOCX) [file pone.0235932.s009.docx]

S7 Table. Correlations between seedling traits and RAF composition of seedlings harvested two years after outplanting in Arctic tundra. Bold indicates significant variables.

| Variable | r^2^ | | p-value | |  |
| --- | --- | --- | --- | --- | --- |
| Biomass (g) | | 0.08 | | 0.08 | |
| **Growth height year 1** | | 0.15 | | 0.01 | |
| Growth height year 2 | | 0.02 | | 0.63 | |
| **Foliar % N** | | 0.47 | | 0.00 | |
| **Foliar % C** | | 0.12 | | 0.04 | |
| **Foliar δ15N** | | 0.18 | | 0.00 | |
| Foliar δ13C | | 0.07 | | 0.16 | |
| Respiration (umol m-2 s-1) | | 0.03 | | 0.43 | |
| Photosynthesis (umol m-2 s-1) | | 0.06 | | 0.2 | |
| **Transpiration (mol m-2 s-1)** | | 0.11 | | 0.05 | |
| Carbon Use Efficiency | | 0.03 | | 0.42 | |
| Δ15N | | 0.04 | | 0.37 | |
| **Δ13C** | | 0.1 0 | | 0.05 | |
